# Supplementary figures and images for: Immunotherapy-induced antibodies to endogenous retroviral envelope glycoprotein confer tumor protection in mice
Source: PLoS One. 2021 Apr 15;16(4):e0248903. doi: 10.1371/journal.pone.0248903 (PMC8049297; doi:10.1371/journal.pone.0248903)

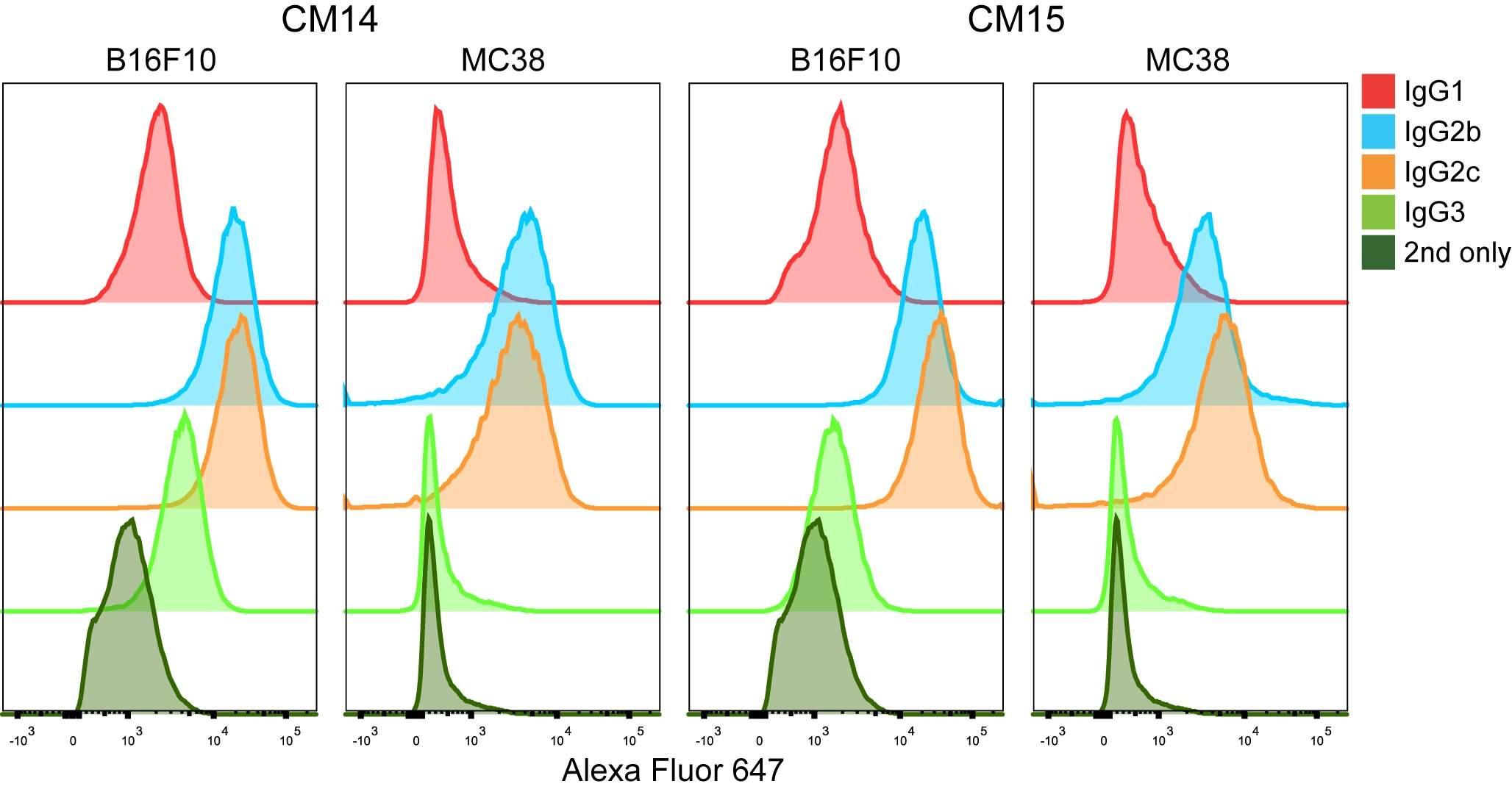

Supplement: S1 Fig — B16F10 and MC38 cells were incubated with indicated sera, stained with Alexa-Fluor-647-conjugated monoclonal Abs against different mouse IgG isotype, and analyzed by flow cytometry. (TIF) [file pone.0248903.s001.tif]

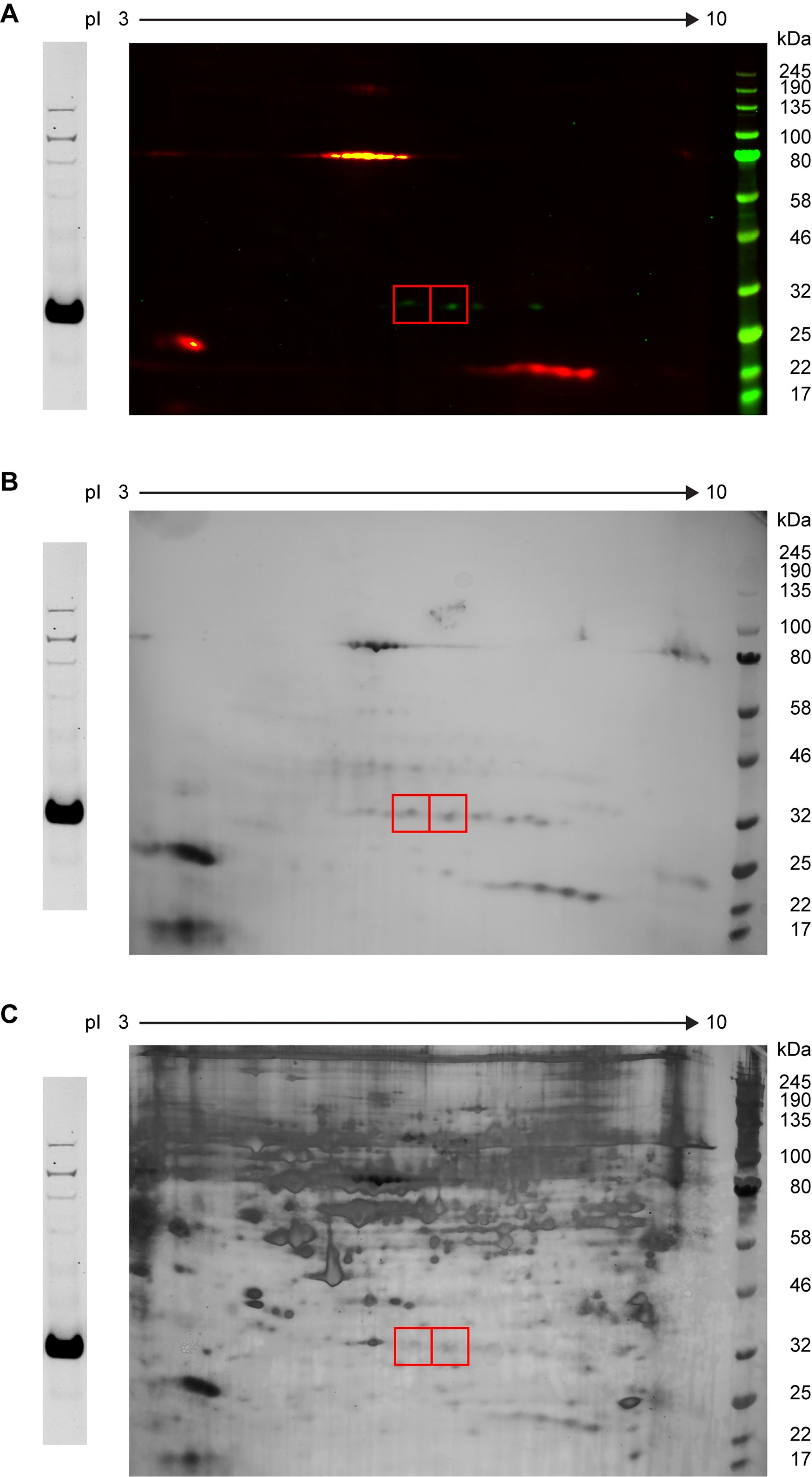

Supplement: S2 Fig — (A) MC38 membrane proteins and biotinylated BSA, soybean trypsin inhibitor, and equine hemoglobin were immunoblotted with CM7 serum and detected with IRDye-800CW-conjugated anti-mouse IgG2b and IgG2c Abs and Alexa-Fluor-647-conjugated streptavidin. (B) Identical sample in (A) was partially transferred and chromogenically detected with biotinylated anti-mouse IgG2b and IgG2c Abs and HRP-conjugated streptavidin. (C) The gel remaining after the partial transfer was silver-stained and overlaid on the chromogenic immunoblot. Red boxes indicate the spots of interest. (TIF) [file pone.0248903.s002.tif]

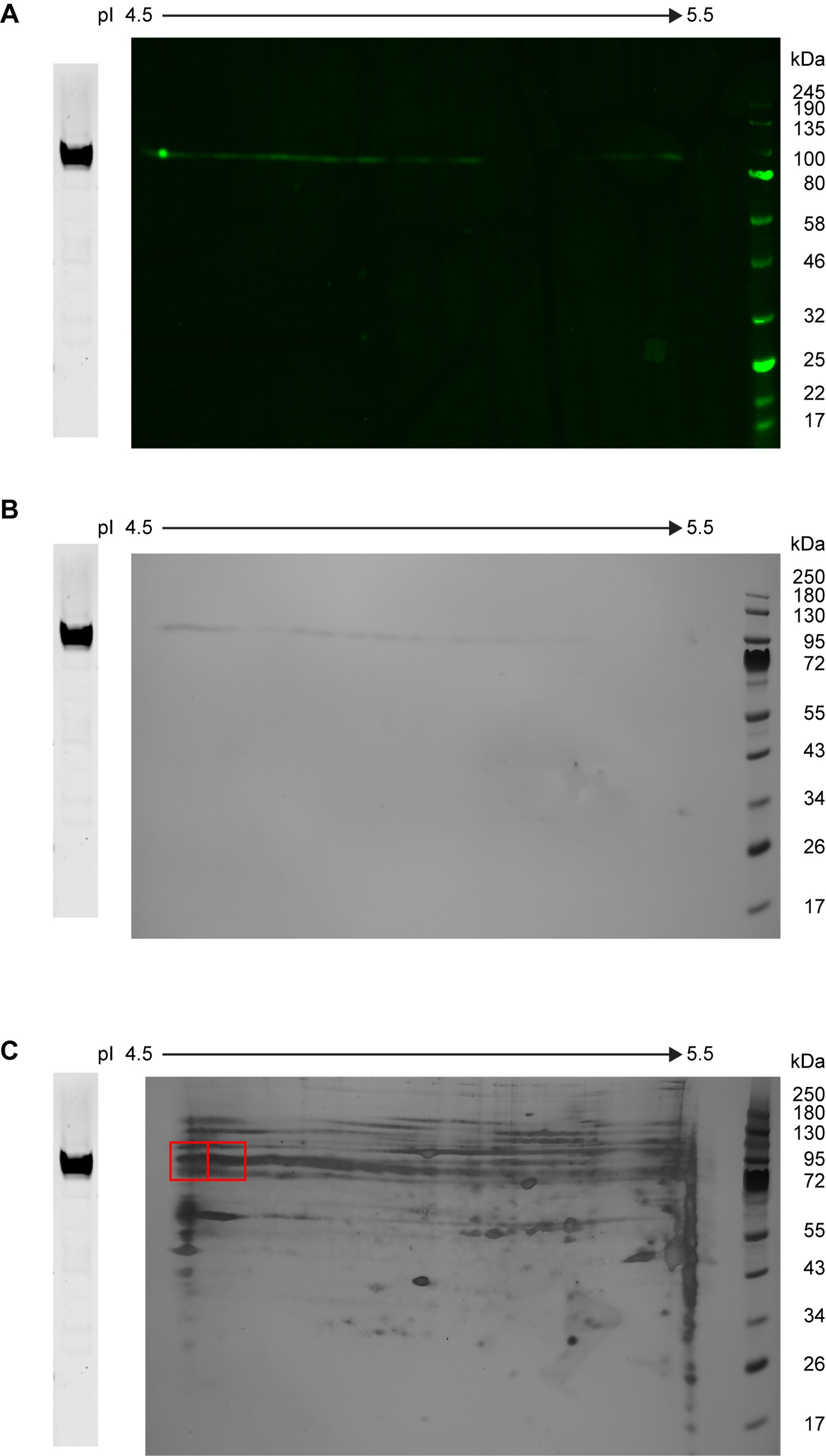

Supplement: S3 Fig — (A) B16F10 membrane proteins were immunoblotted with CM6 serum and detected with IRDye-800CW-conjugated anti-mouse IgG2b and IgG2c Abs. (B) Identical sample in (A) was partially transferred and chromogenically detected with biotinylated anti-mouse IgG2b and IgG2c Abs and HRP-conjugated streptavidin. (C) The gel remaining after the partial transfer was silver-stained and overlaid on the chromogenic immunoblot. Red boxes indicate the spots of interest. (TIF) [file pone.0248903.s003.tif]

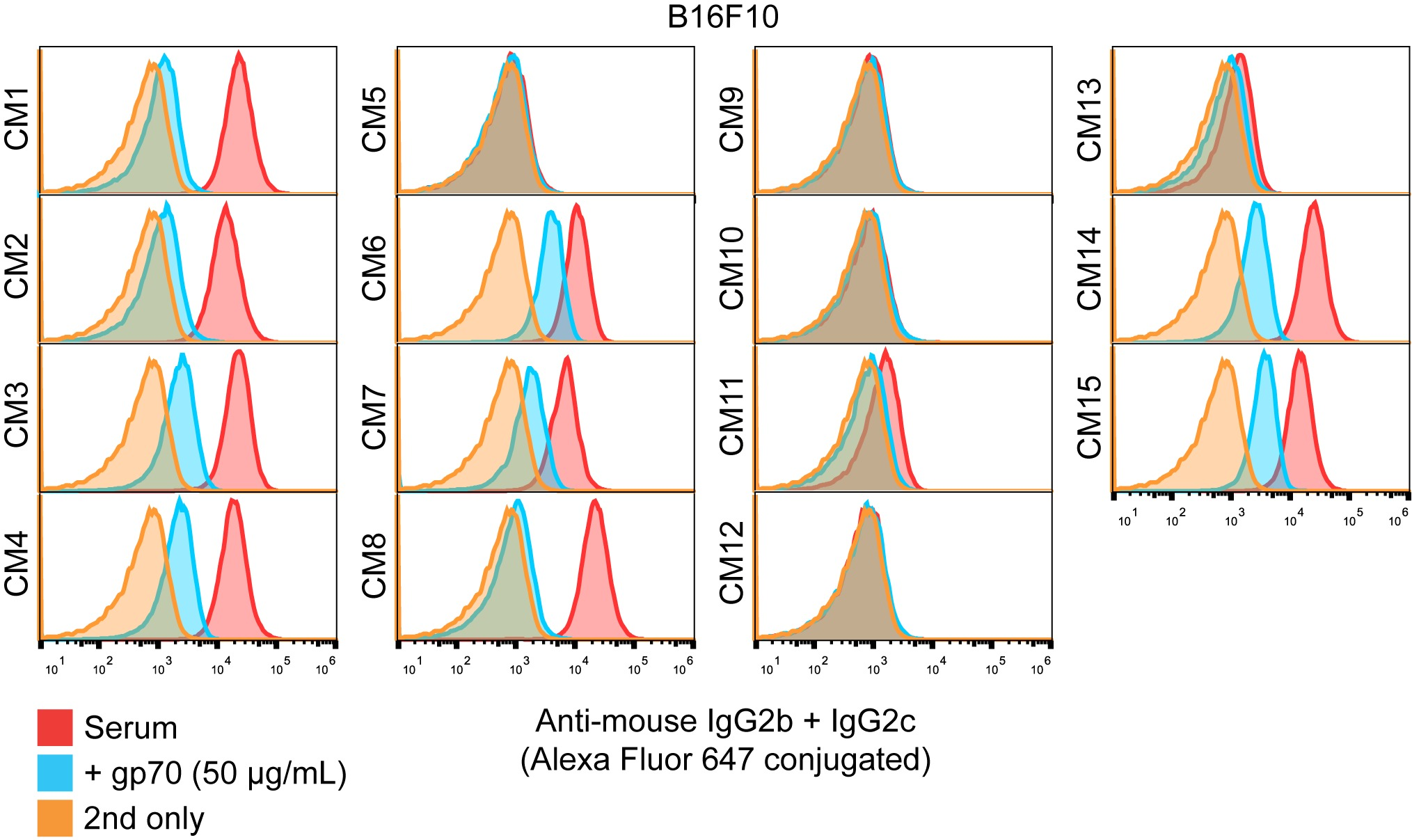

Supplement: S4 Fig — B16F10 cells were incubated with 1% serum or 10 μg/ml TA99 in the presence of 50 μg/ml gp70 and analyzed as in Fig 3B. (TIF) [file pone.0248903.s004.tif]

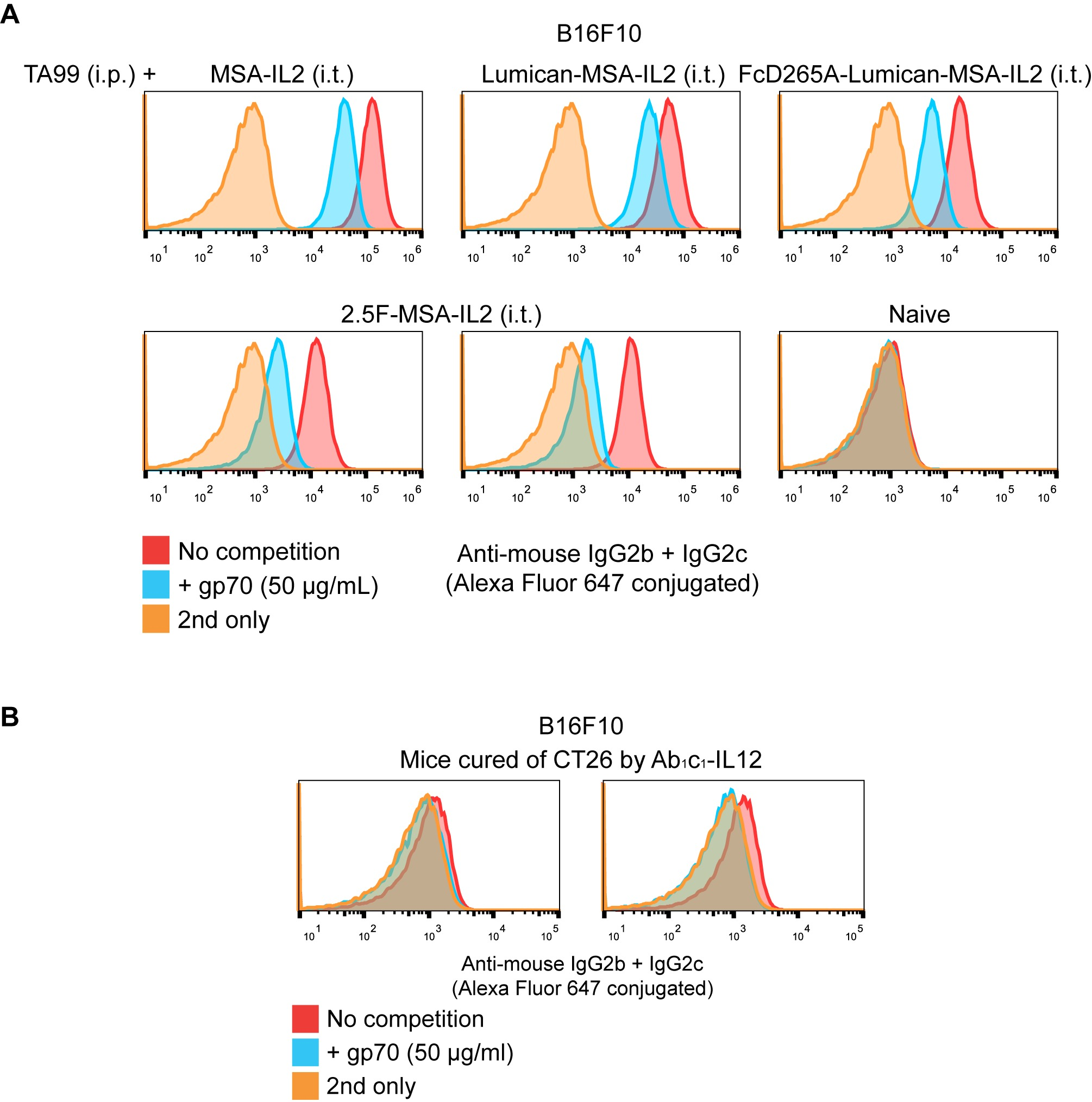

Supplement: S5 Fig — (A,B) B16F10 cells were incubated with 1% serum collected from mice that received indicated immunotherapies in the presence of 50 μg/ml gp70 and analyzed as in Fig 3B. (TIF) [file pone.0248903.s005.tif]

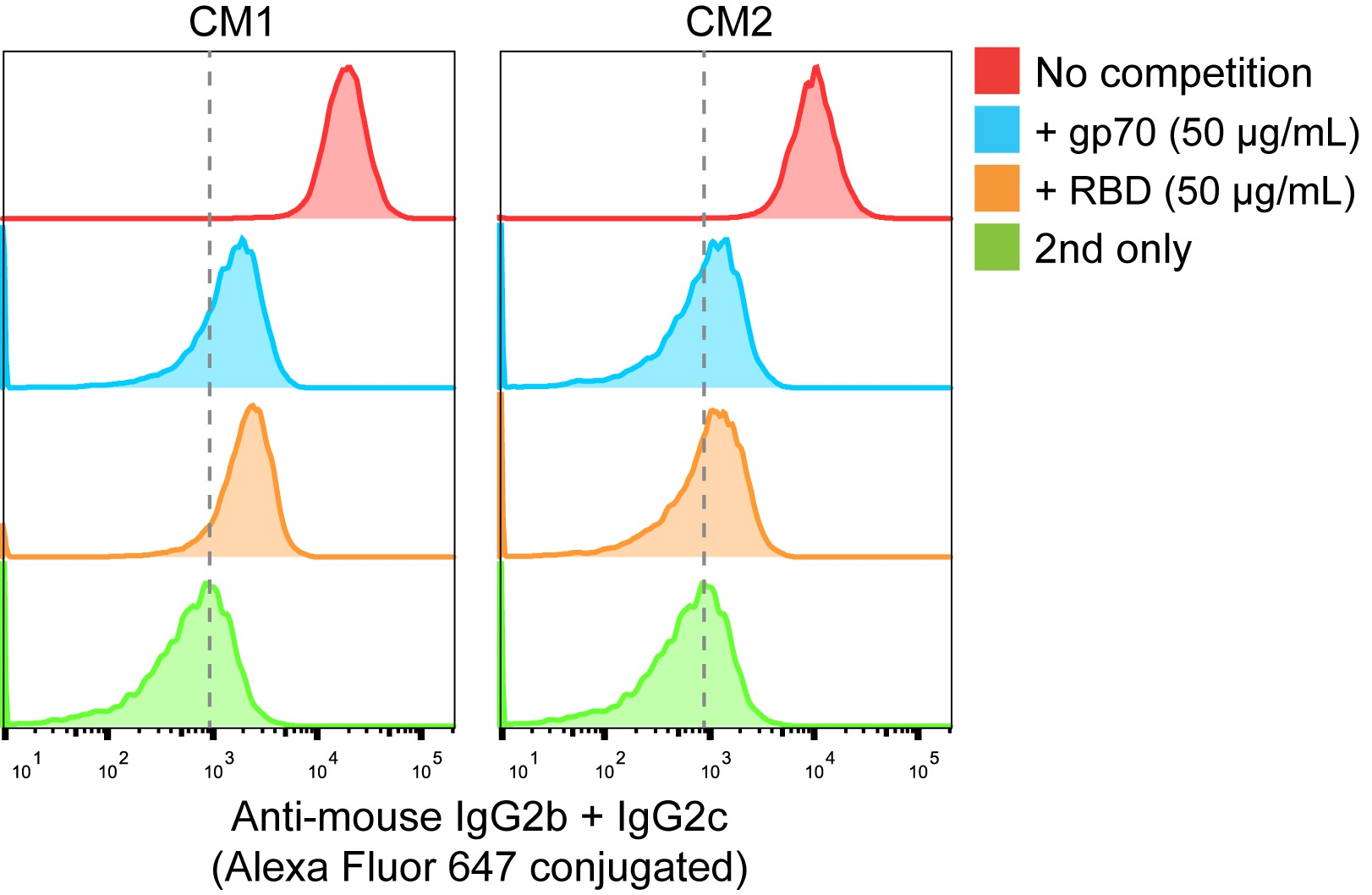

Supplement: S6 Fig — B16F10 cells were incubated with 1% serum in the presence of 50 μg/ml gp70 or RBD and analyzed as in Fig 3B. (TIF) [file pone.0248903.s006.tif]

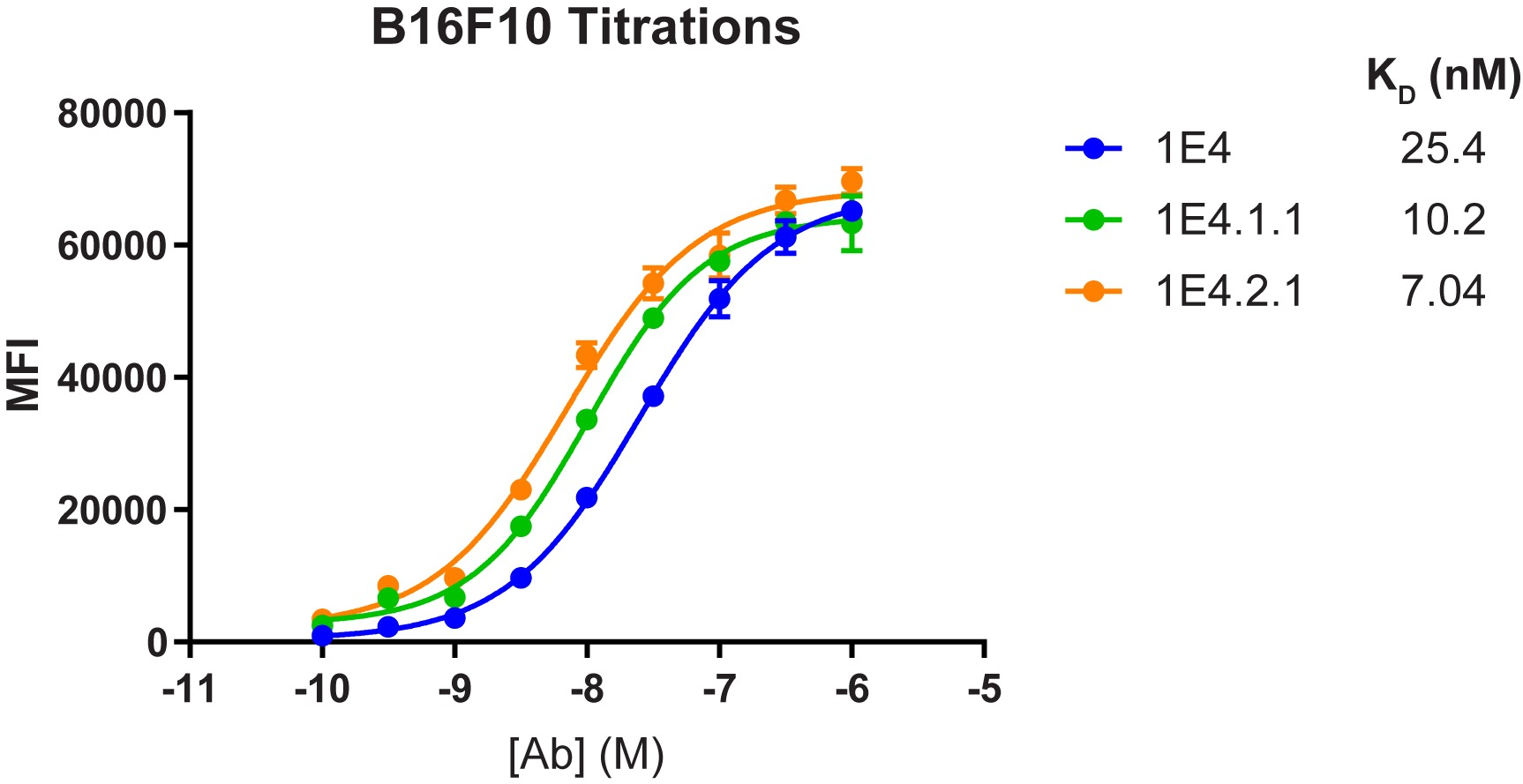

Supplement: S7 Fig — B16F10 cells were incubated with soluble 1E4 and affinity-matured clones at indicated concentrations and analyzed as in Fig 1A. (TIF) [file pone.0248903.s007.tif]

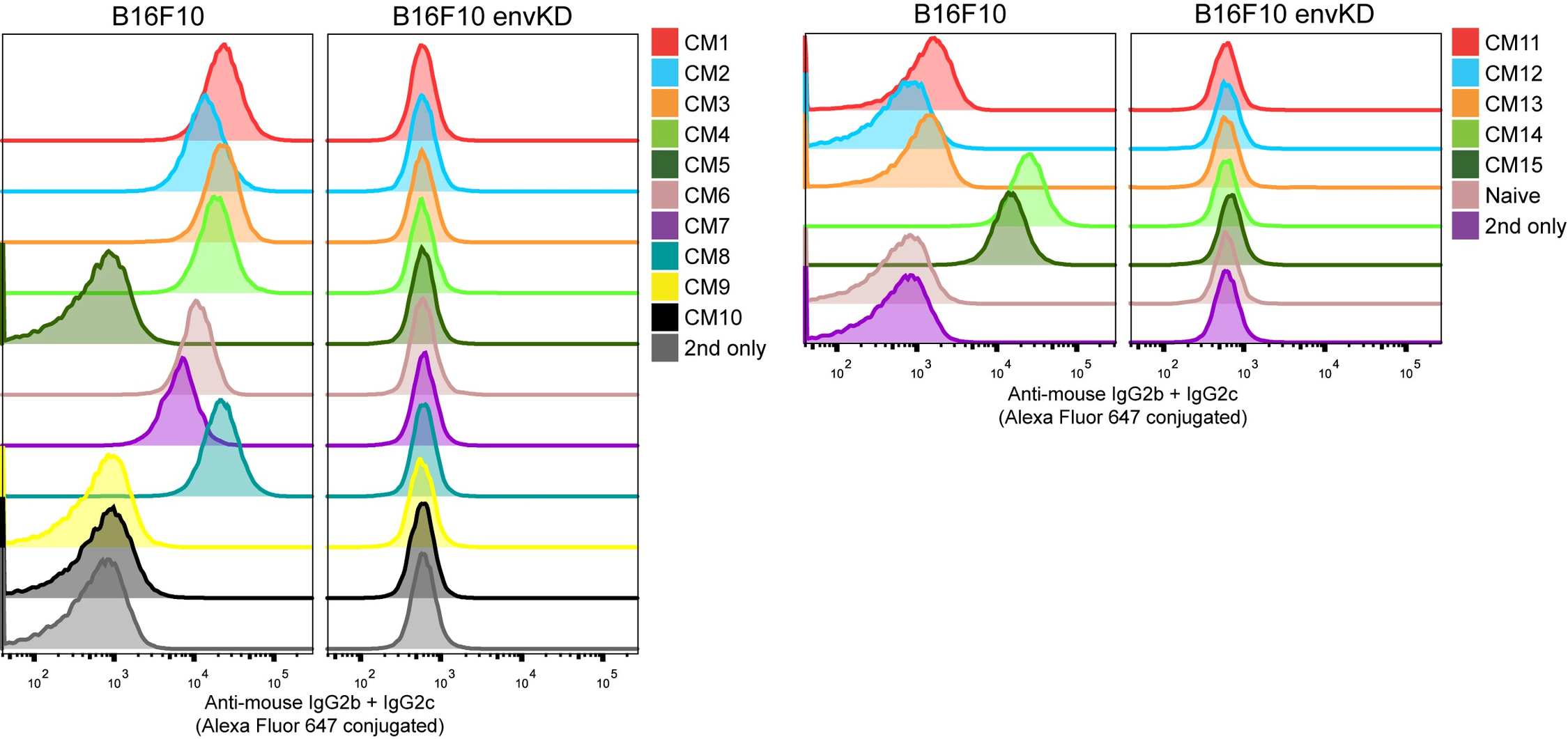

Supplement: S8 Fig — B16F10 or B16F10 envKO cells were incubated with 1% serum and analyzed as in Fig 1A. (TIF) [file pone.0248903.s008.tif]

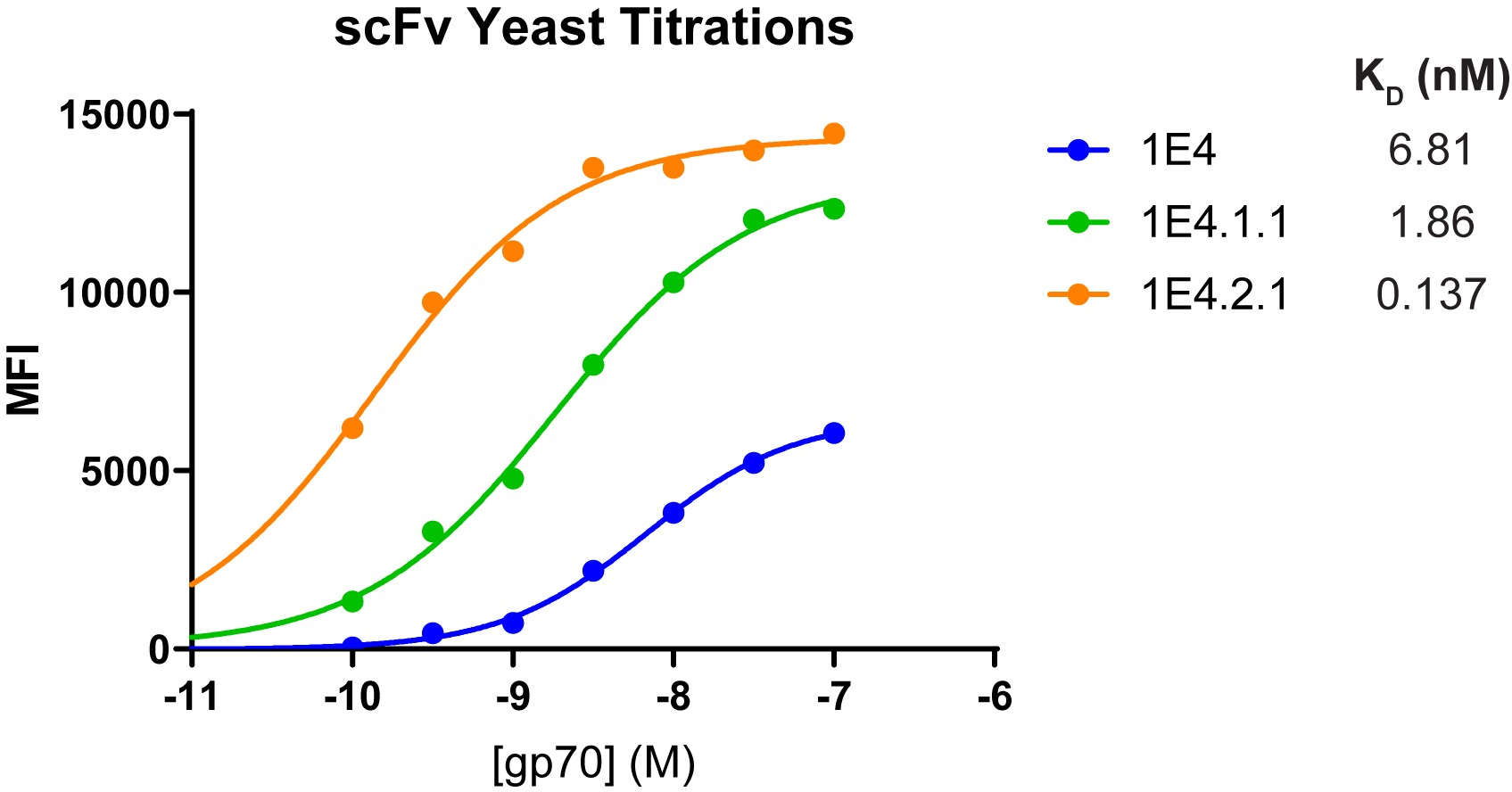

Supplement: S9 Fig — Yeast cells expressing scFv’s of 1E4 and affinity-matured clones were were incubated with chicken anti-c-myc Ab and biotinylated gp70 at indicated concentrations, washed, stained with Alexa-Fluor-488-conjugated streptavidin and Alexa-Fluor-647-conjugated anti-chicken IgG secondary Ab, and analyzed by flow cytometry. (TIF) [file pone.0248903.s009.tif]

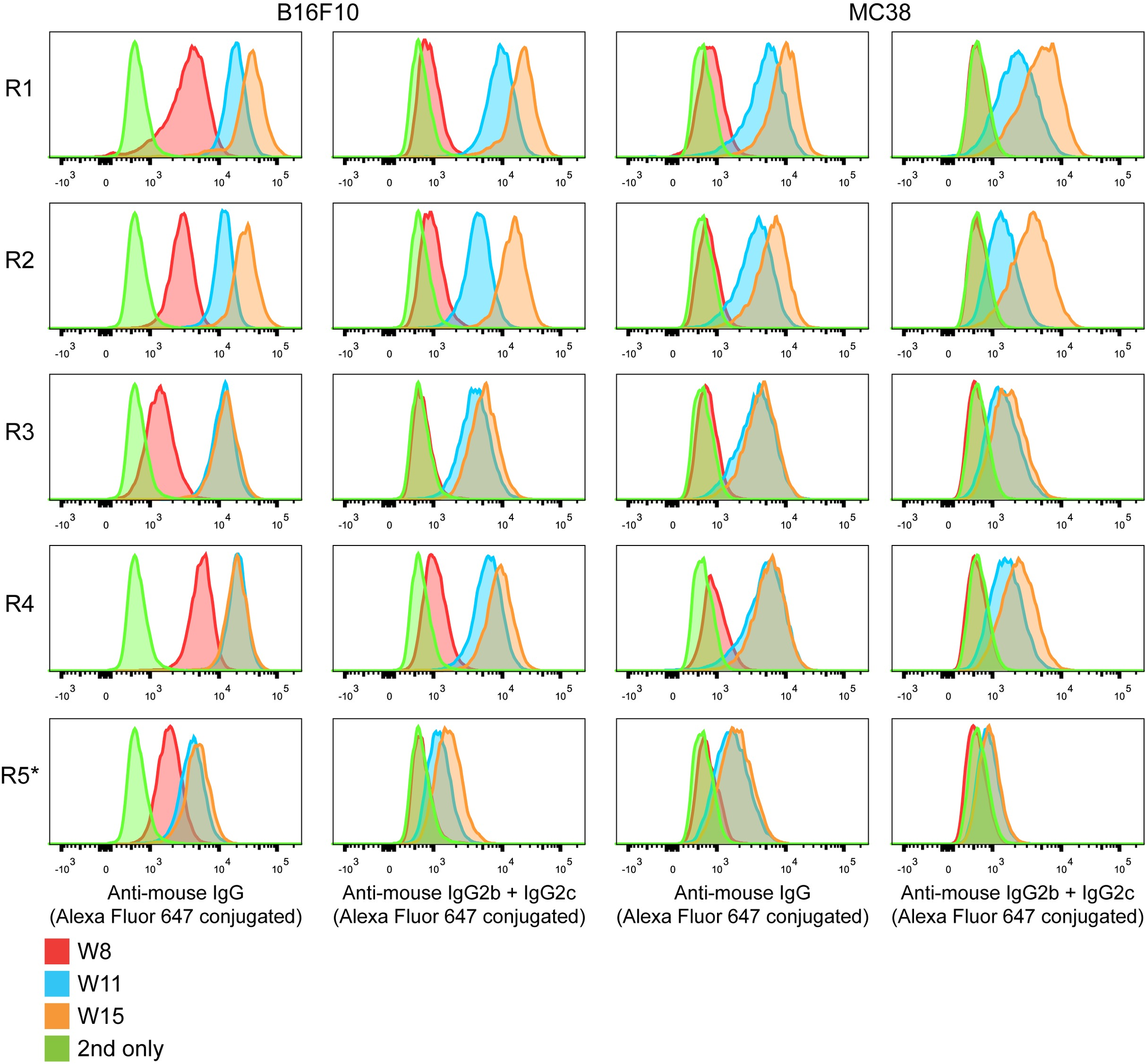

Supplement: S10 Fig — Naive mice were vaccinated on weeks 0, 3, 6, 9, and 12 with RBD adjuvanted with saponin-MPLA nanoparticles (R# for each RBD-vaccinated mouse) and serum was collected on indicated weeks. B16F10 and MC38 cells were incubated with 1% serum, stained with Alexa-Fluor-647-conjugated anti-mouse IgG or IgG2b and IgG2c, and analyzed by flow cytometry. *Asterisk denotes the mouse that succumbed to the tumor challenge. (TIF) [file pone.0248903.s010.tif]

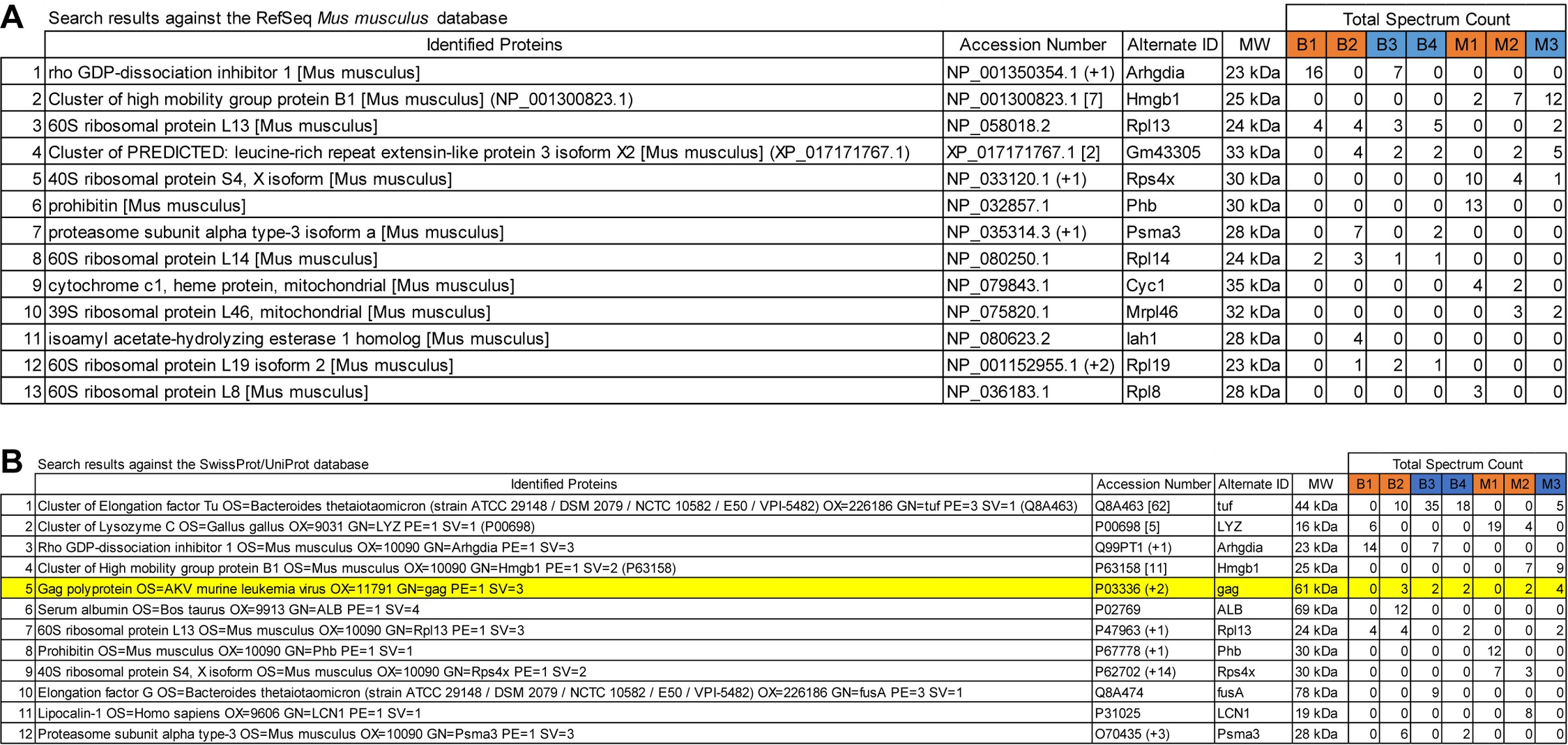

Supplement: S1 Table — lue colors indicate independent experiments. Highlighted row indicates the antigen identified. (TIF) [file pone.0248903.s011.tif]

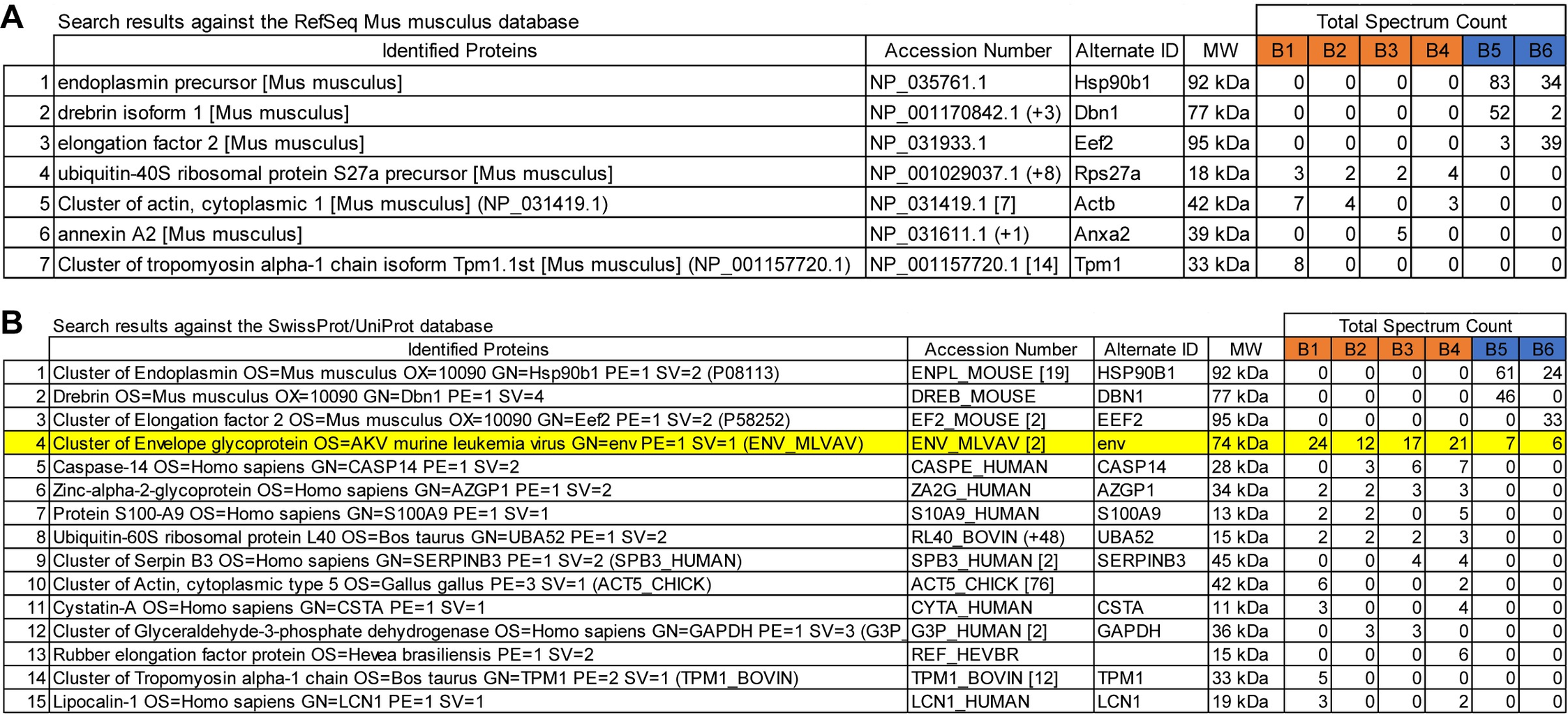

Supplement: S2 Table — Data was searched against the (A) RefSeq Mus musculus database and (B) SwissProt/UniProt database. Orange and blue colors indicate independent experiments. Highlighted row indicates the antigen identified. (TIF) [file pone.0248903.s012.tif]
