# Supplementary material for: Immunotherapy-induced antibodies to endogenous retroviral envelope glycoprotein confer tumor protection in mice
Source: PLoS One. 2021 Apr 15;16(4):e0248903. doi: 10.1371/journal.pone.0248903 (PMC8049297; doi:10.1371/journal.pone.0248903)
Supplement: S1 Raw images — (PDF) [file pone.0248903.s013.pdf]

Raw images for Fig 1D

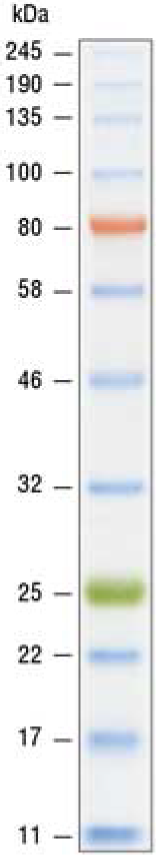

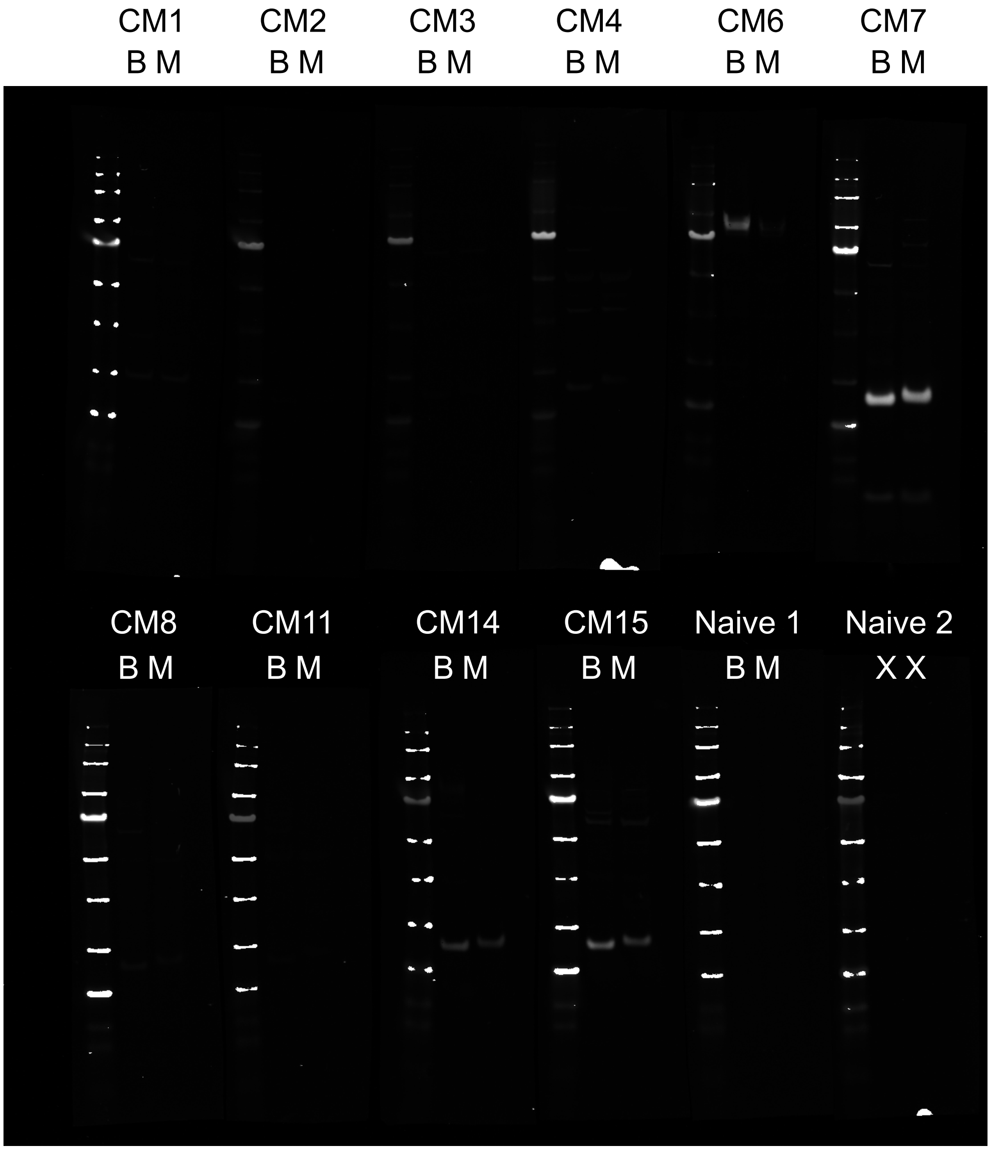

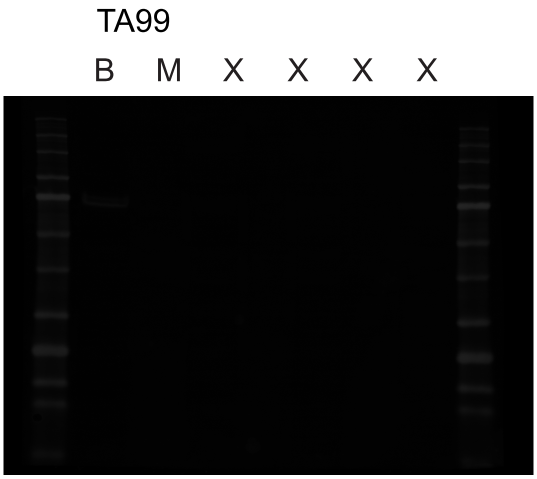

B = B16F10 membrane proteins
M = MC38 membrane proteins

Scanned with LI-COR Odyssey 9120 Imager in 800 nm channel

Raw images for Fig 2B

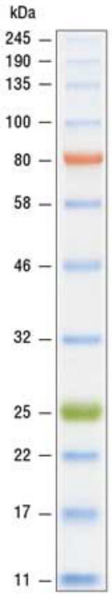

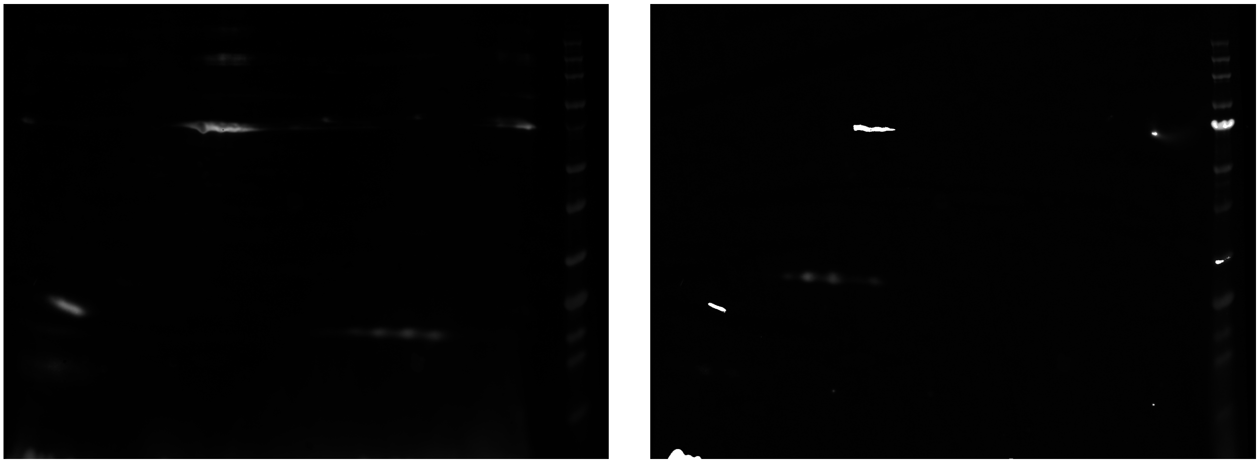

B16F10 membrane proteins and streptavidin-conjugated control proteins were probed with CM7 serum. The immunoblot was scanned with LI-COR Odyssey 9120 Imager in 700 nm (left) or 800 nm (right) channel.

Raw images for Fig 2C,D

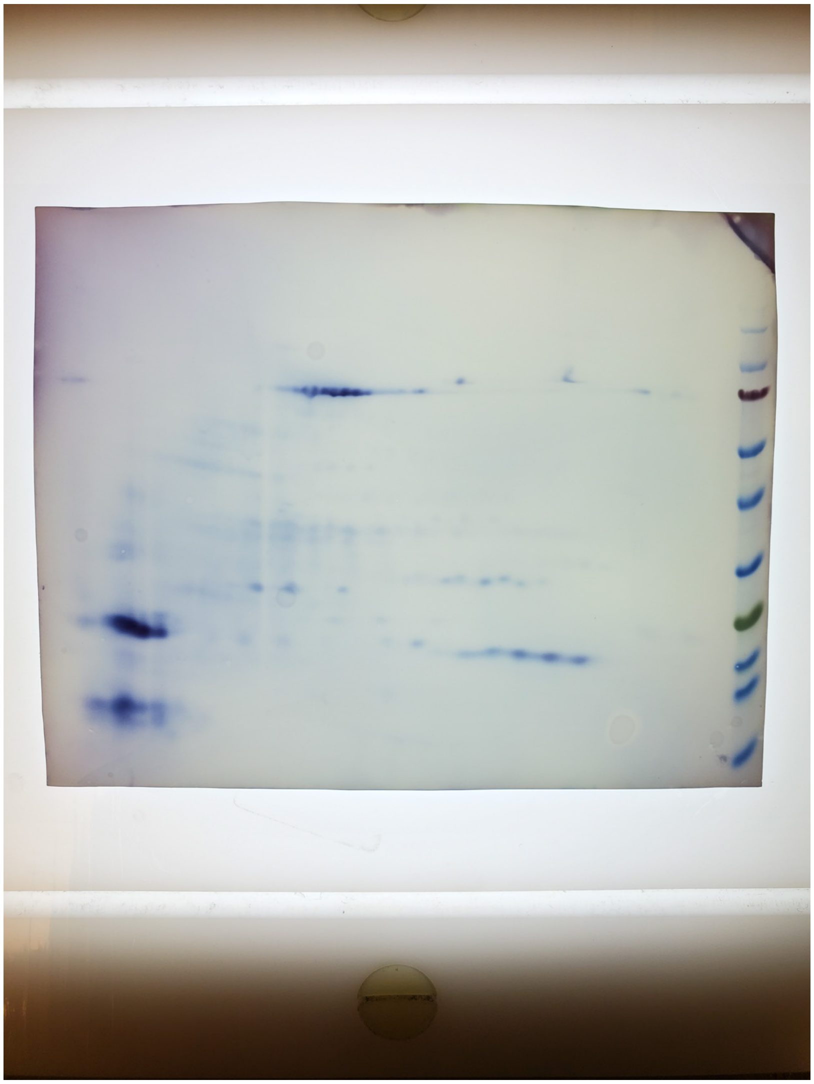

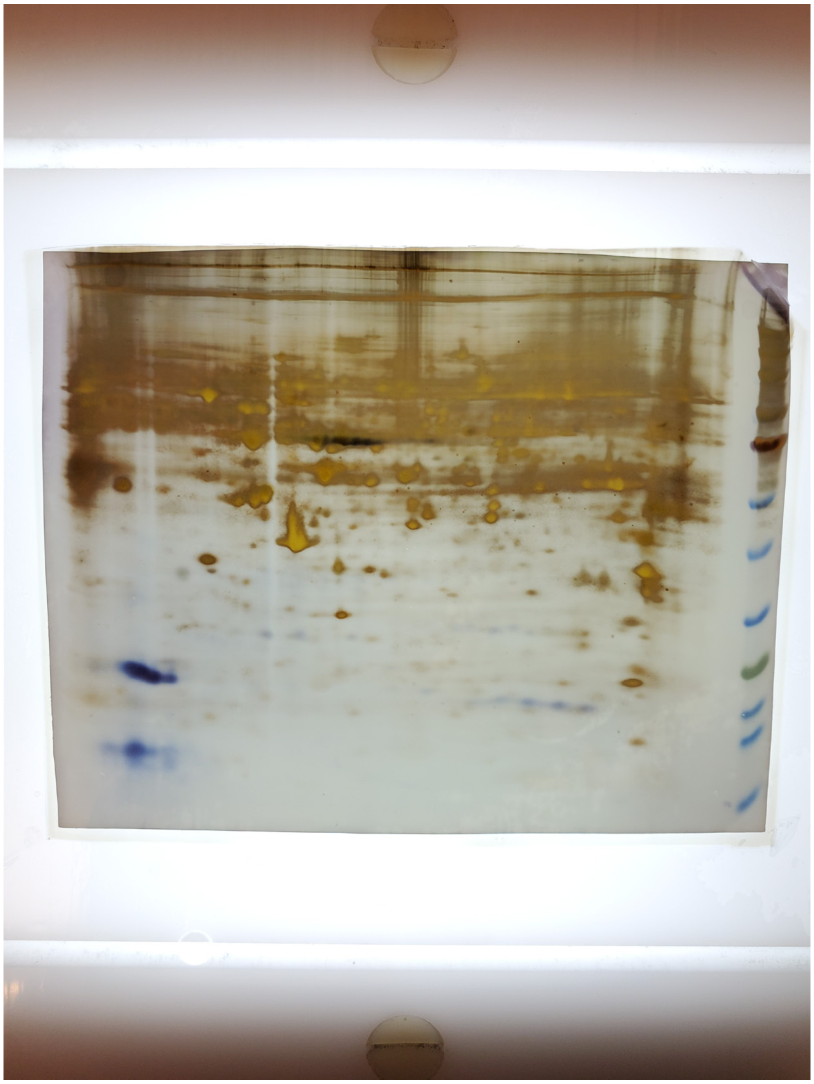

B16F10 membrane proteins and streptavidin-conjugated control proteins were probed with CM7 serum. The immunoblot and the gel were imaged with Samsung SM-G973U.

Raw images for Fig 3A

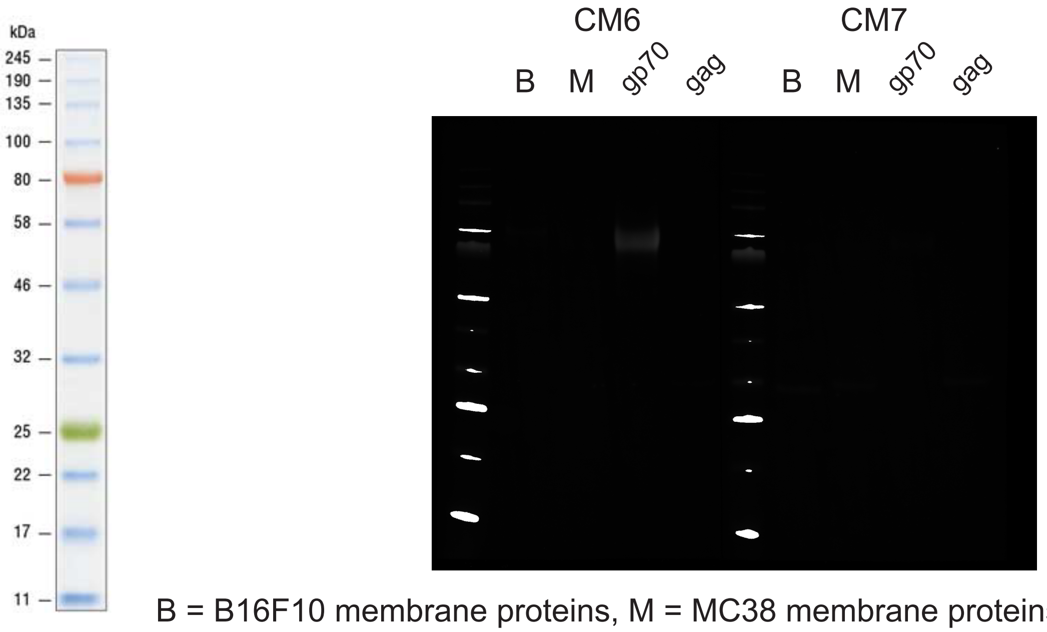

B = B16F10 membrane proteins, M = MC38 membrane proteins
Indicated samples were probed with CM6 (left) and CM7 (right) serum.
The immunoblot was scanned with LI-COR Odyssey 9120 Imager in 800 nm channel.

Raw images for S2A Fig

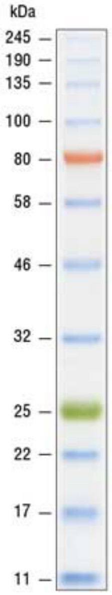

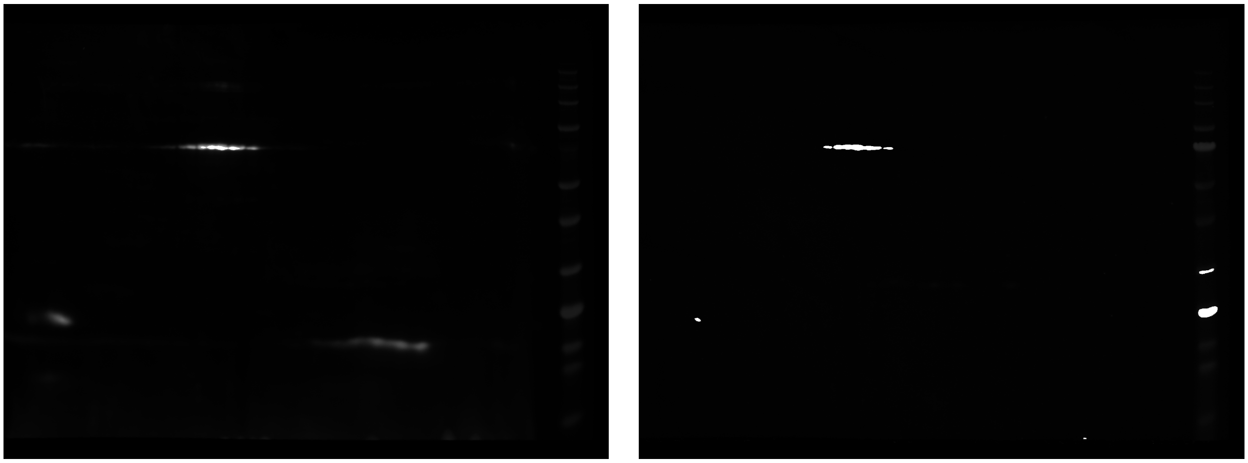

MC38 membrane proteins and streptavidin-conjugated control proteins were probed with CM7 serum. The immunoblot was scanned with LI-COR Odyssey 9120 Imager in 700 nm (left) or 800 nm (right) channel.

Raw images for S2B,C Fig

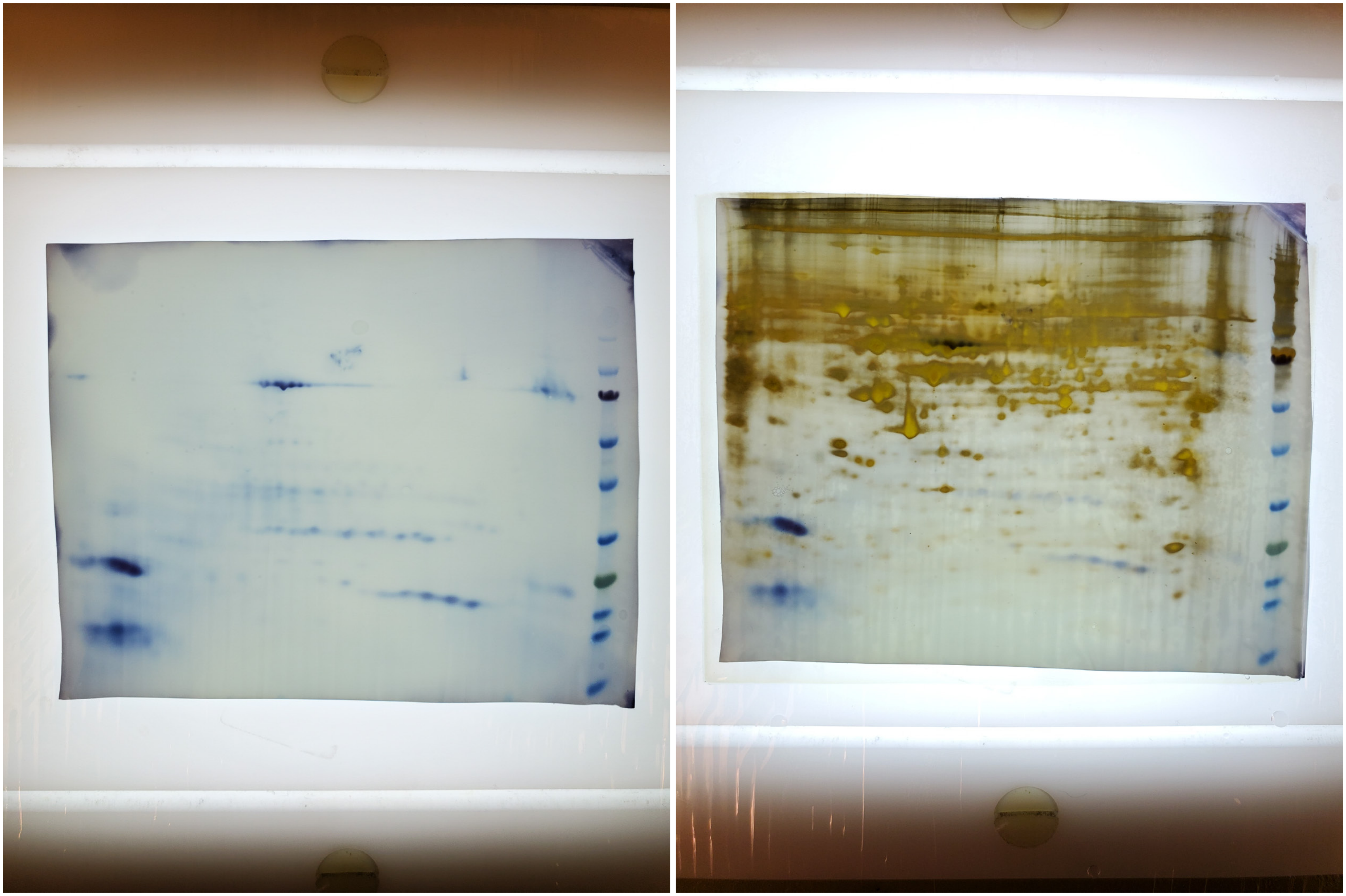

MC38 membrane proteins and streptavidin-conjugated control proteins were probed with CM7 serum. The immunoblot and the gel were imaged with Samsung SM-G973U.

Raw images for S3A Fig

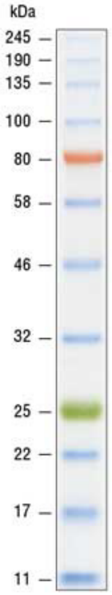

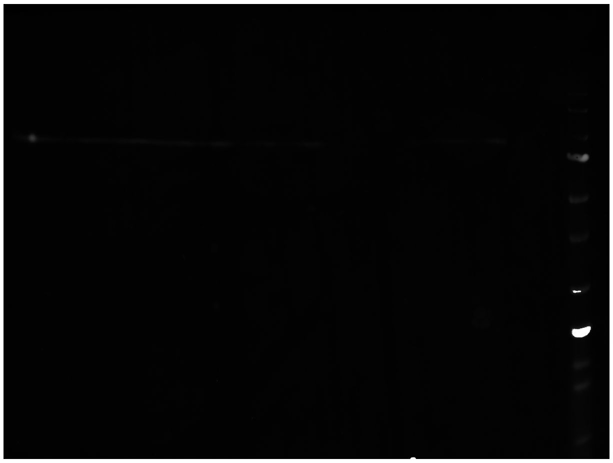

B16F10 membrane proteins and streptavidin-conjugated control proteins were probed with CM6 serum. The immunoblot was scanned with LI-COR Odyssey 9120 Imager in 800 nm channel.

Raw images for S3B,C Fig

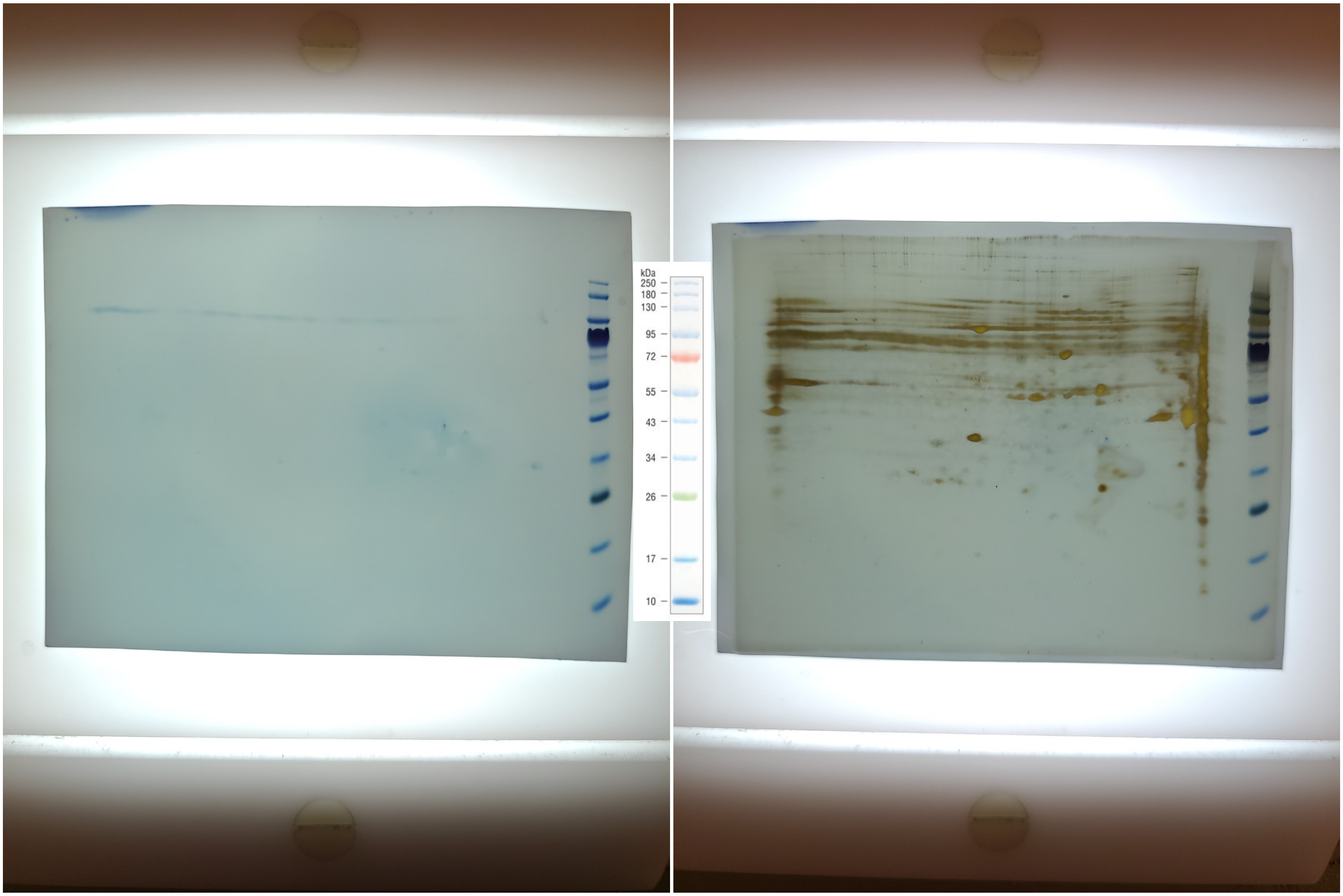

B16F10 membrane proteins and streptavidin-conjugated control proteins were probed with CM6 serum. The immunoblot and the gel were imaged with Samsung SM-G981U.
