# Supplementary material for: Immunotherapy-induced antibodies to endogenous retroviral envelope glycoprotein confer tumor protection in mice
Source: PLoS One. 2021 Apr 15;16(4):e0248903. doi: 10.1371/journal.pone.0248903 (PMC8049297; doi:10.1371/journal.pone.0248903)
Supplement: Attachment — Submitted filename: response to reviewers.docx [file pone.0248903.s014.docx]

January 23, 2021

Re: PLOS One manuscript PONE-D-20-33132

Dear Editors,

We are uploading a revised version of PONE-D-20-33132, “Immunotherapy-induced antibodies to endogenous retroviral envelope glycoprotein confer tumor protection in mice”, which we believe addresses the suggested changes. Detailed responses follow in this letter, in bold-faced red.

Sincerely,

K. Dane Wittrup

C.P. Dubbs Professor

Chemical & Biological Engineering

1. Please ensure that your manuscript meets PLOS ONE's style requirements, including those for file naming. The PLOS ONE style templates can be found at

[https://journals.plos.org/plosone/s/file?id=wjVg/PLOSOne_formatting_sample_main_body.pdf](https://www.google.com/url?q=https://journals.plos.org/plosone/s/file?id%3DwjVg/PLOSOne_formatting_sample_main_body.pdf&source=gmail-imap&ust=1611351457000000&usg=AOvVaw339xbxpvivktFDN7M9ysXj) and

[https://journals.plos.org/plosone/s/file?id=ba62/PLOSOne_formatting_sample_title_authors_affiliations.pdf](https://www.google.com/url?q=https://journals.plos.org/plosone/s/file?id%3Dba62/PLOSOne_formatting_sample_title_authors_affiliations.pdf&source=gmail-imap&ust=1611351457000000&usg=AOvVaw1MTYoi3L8j3qGyxTMSR9vG)

**The manuscript has been revised to meet the style requirements.**

2.PLOS ONE now requires that authors provide the original uncropped and unadjusted images underlying all blot or gel results reported in a submission’s figures or Supporting Information files. This policy and the journal’s other requirements for blot/gel reporting and figure preparation are described in detail at [https://journals.plos.org/plosone/s/figures#loc-blot-and-gel-reporting-requirements](https://www.google.com/url?q=https://journals.plos.org/plosone/s/figures%23loc-blot-and-gel-reporting-requirements&source=gmail-imap&ust=1611351457000000&usg=AOvVaw3M_LuBJk9Exk89w1-7N4Ra) and [https://journals.plos.org/plosone/s/figures#loc-preparing-figures-from-image-files](https://www.google.com/url?q=https://journals.plos.org/plosone/s/figures%23loc-preparing-figures-from-image-files&source=gmail-imap&ust=1611351457000000&usg=AOvVaw20xSR4ENJUMm_r1RXEPh7j). When you submit your revised manuscript, please ensure that your figures adhere fully to these guidelines and provide the original underlying images for all blot or gel data reported in your submission. See the following link for instructions on providing the original image data: [https://journals.plos.org/plosone/s/figures#loc-original-images-for-blots-and-gels](https://www.google.com/url?q=https://journals.plos.org/plosone/s/figures%23loc-original-images-for-blots-and-gels&source=gmail-imap&ust=1611351457000000&usg=AOvVaw26kTyi1bnPod1IhjSePrpt).

In your cover letter, please note whether your blot/gel image data are in Supporting Information or posted at a public data repository, provide the repository URL if relevant, and provide specific details as to which raw blot/gel images, if any, are not available. Email us at [plosone@plos.org](mailto:plosone@plos.org) if you have any questions.

**The original images are compiled in the S1_raw_images.pdf file. They are for Figs 1D, 2B, 2C, 2D, 3A, S2 and S3.**

3. We note that you have included the phrase “data not shown” in your manuscript. Unfortunately, this does not meet our data sharing requirements. PLOS does not permit references to inaccessible data. We require that authors provide all relevant data within the paper, Supporting Information files, or in an acceptable, public repository. Please add a citation to support this phrase or upload the data that corresponds with these findings to a stable repository (such as Figshare or Dryad) and provide and URLs, DOIs, or accession numbers that may be used to access these data. Or, if the data are not a core part of the research being presented in your study, we ask that you remove the phrase that refers to these data.

**The phrase was removed from the manuscript.**

Additional Editor Comments:

Introduction

and immunosurveillance[1,2].

Please chasnge to

and immunosurveillance [1,2].

Please insert space before the reference numbers throughout the manuscript.

**Revised.**

[Note: HTML markup is below. Please do not edit.]

Reviewers' comments:

Reviewer's Responses to Questions

**Comments to the Author**

1. Is the manuscript technically sound, and do the data support the conclusions?

The manuscript must describe a technically sound piece of scientific research with data that supports the conclusions. Experiments must have been conducted rigorously, with appropriate controls, replication, and sample sizes. The conclusions must be drawn appropriately based on the data presented.

Reviewer #1: Yes

Reviewer #2: Yes

2. Has the statistical analysis been performed appropriately and rigorously?

Reviewer #1: Yes

Reviewer #2: Yes

3. Have the authors made all data underlying the findings in their manuscript fully available?

The [PLOS Data policy](https://www.google.com/url?q=http://www.plosone.org/static/policies.action%23sharing&source=gmail-imap&ust=1611351457000000&usg=AOvVaw168cWx73EOLVHNZozzoF3i) requires authors to make all data underlying the findings described in their manuscript fully available without restriction, with rare exception (please refer to the Data Availability Statement in the manuscript PDF file). The data should be provided as part of the manuscript or its supporting information, or deposited to a public repository. For example, in addition to summary statistics, the data points behind means, medians and variance measures should be available. If there are restrictions on publicly sharing data—e.g. participant privacy or use of data from a third party—those must be specified.

Reviewer #1: Yes

Reviewer #2: Yes

4. Is the manuscript presented in an intelligible fashion and written in standard English?

PLOS ONE does not copyedit accepted manuscripts, so the language in submitted articles must be clear, correct, and unambiguous. Any typographical or grammatical errors should be corrected at revision, so please note any specific errors here.

Reviewer #1: Yes

Reviewer #2: Yes

5. Review Comments to the Author

Please use the space provided to explain your answers to the questions above. You may also include additional comments for the author, including concerns about dual publication, research ethics, or publication ethics. (Please upload your review as an attachment if it exceeds 20,000 characters)

Reviewer #1: Kang et al. describe the identification of the mouse tumor antigen underlying a humoral response and tumor challenge response, Env glycoprotein. The study generally was performed well with appropriate rigor to guide the conclusions. The paper will be of interest to others in the oncology field and the discussion does an excellent job capturing the impact (although not key to Plos One) of the article. I have a few minor concerns that should be addressed to help improve the manuscript prior to publication.

Flow figures are completely lacking quantification- although this allows qualitative claims, authors mention things like "we conclusively demonstrated that eMLV env is the primary cell-surface antigen...." the manuscript would help with quantitation to support such statements.

**A new panel (Fig 3D) was added to quantitate the significant change in MFI of B16F10 WT vs envKD cells stained with the antisera.**

Figure 1 legend gives statistical confidence ranges ** and *** that were not used in the figure as far as I could tell.

**Unused ones were removed.**

Figure 1D- were all 10 CM samples from cured mice that were successful in rejecting the secondary challenge? same or different mice that those 5 shown in 1C?

**All CM mice had rejected a secondary challenge. This was clarified in the text by changing "sera from cured mice" to "sera from cured mice that rejected a secondary challenge."**

Figure 2B- indicate the colors found on the blot in the legend that correspond to IR800 and AF-647.

**The legend was revised to indicate the colors.**

Env and GP70 are used somewhat interchangeably (referring to protein and gene) and could create confusion, moreover later in the paper monomeric versus trimeric possibilities are introduced. The authors should stick with one naming convention throughout the text and figures. They should also be clear about what protein (monomeric GP70?) is being used for flow assays, competition, affinity maturation and vaccination.

**We intended italicized *env* to refer to the gene and non-italicized env to refer to the envelope glycoprotein made up of gp70 and p15E subunit proteins. The text has been revised to show gp70 only when solubly expressed gp70 was used in the experiment and where applicable, monomeric nature of gp70 was explicitly stated to avoid confusion.**

Reviewer #2: In this study, Kang et al studied the roles of antibodies against glycoproteins from murine ERV in immunotherapy and discovered their key roles in mediating anti-tumor effect during cancer immunotherapy. The study was based on previous studies in the Witrrup lab showing that in mice cured of their tumors by different immunotherapeutic methods, antibodies against tumor cell surface antigens played critical roles. The authors adopted a systematic approach and was able to identify eMLV gp70 as the commons murine tumor antigen that was shared among different tumor lines. Of particular interest is the finding that antibodies against this antigen had potent anti-tumor effect against multiple syngeneic or allogenic tumor cell line. The study itself is well designed and carried out in a very competent manner. More importantly, it has significant implications for human cancer treatment since ERV expression has been observed in many human tumors as well.

6. PLOS authors have the option to publish the peer review history of their article ([what does this mean?](https://www.google.com/url?q=https://journals.plos.org/plosone/s/editorial-and-peer-review-process%23loc-peer-review-history&source=gmail-imap&ust=1611351457000000&usg=AOvVaw0c2ZyG2z5tzzrTvPf6kNiT)). If published, this will include your full peer review and any attached files.


If you choose “no”, your identity will remain anonymous but your review may still be made public.


**Do you want your identity to be public for this peer review?** For information about this choice, including consent withdrawal, please see our [Privacy Policy](https://www.google.com/url?q=https://www.plos.org/privacy-policy&source=gmail-imap&ust=1611351457000000&usg=AOvVaw3lDZCsFHl0cAWhiyxexImm).

Reviewer #1: No

Reviewer #2: No

[NOTE: If reviewer comments were submitted as an attachment file, they will be attached to this email and accessible via the submission site. Please log into your account, locate the manuscript record, and check for the action link "View Attachments". If this link does not appear, there are no attachment files.]

While revising your submission, please upload your figure files to the Preflight Analysis and Conversion Engine (PACE) digital diagnostic tool, [https://pacev2.apexcovantage.com/](https://www.google.com/url?q=https://pacev2.apexcovantage.com/&source=gmail-imap&ust=1611351457000000&usg=AOvVaw3qF6JvxiRpNHQF2fGjWhnD). PACE helps ensure that figures meet PLOS requirements. To use PACE, you must first register as a user. Registration is free. Then, login and navigate to the UPLOAD tab, where you will find detailed instructions on how to use the tool. If you encounter any issues or have any questions when using PACE, please email PLOS at [figures@plos.org](mailto:figures@plos.org). Please note that Supporting Information files do not need this step.
